# NSD3, a member of nuclear receptor‐binding SET domain family, is a potential prognostic biomarker for pancreatic cancer

**DOI:** 10.1002/cam4.5774

**Published:** 2023-04-16

**Authors:** Qunli Xiong, Ying Zhou, Su Zhang, Yaguang Zhang, Yongfeng Xu, Yang Yang, Congya Zhou, Zhu Zeng, Junhong Han, Qing Zhu

**Affiliations:** ^1^ Department of Abdominal Oncology, West China Hospital Sichuan University Chengdu China; ^2^ Research Laboratory of Cancer Epigenetics and Genomics, Department of General Surgery, Frontiers Science Center for Disease‐Related Molecular Network, State Key Laboratory of Biotherapy, West China Hospital Sichuan University Chengdu China; ^3^ Department of Radiation Oncology Shaanxi Provincial People's Hospital Xi'an China

**Keywords:** cell proliferation, immune infiltration, NSD3, nuclear receptor‐binding SET domain, pancreatic cancer, prognosis biomarker

## Abstract

**Background:**

Members of the nuclear receptor‐binding SET domain (NSD) family of histone H3 lysine 36 methyltransferases comprise NSD1, NSD2 (MMSET/WHSC1), and NSD3 (Wolf–Hirschhorn syndrome candidate 1‐like 1, WHSC1L1). While the expression of NSD genes is essential to normal biological processes and cancer, knowledge of their expression levels to prognosticate in cancer remains unclear.

**Methods:**

We analyzed the expression patterns for NSD family genes across multiple cancer types and examined their association with clinical features and patient survival profiles. Next, we explored the association between NSD3 expression and described features of the tumor microenvironment (TME) in PAAD, a severe type of pancreatic cancer. In particular, we correlated promoter methylation levels for NSD3 with patient outcomes in PAAD. Finally, we explored the putative oncogenic roles for NSD3 using a series of experiments with pancreatic cancer cells.

**Results:**

We report that the expression of NSD family members is correlated with clinical prognosis across multiple types of cancers. Also, we demonstrate that NSD3 variants are most prevalent among NSD genes across cancers we analyzed. Notably, when compared with NSD1 and NSD2, we find that NSD3 is prominently expressed, and its expression is significantly linked with clinical outcome in pancreatic cancer. Furthermore, *NSD3* is frequently amplified, exhibits low promoter methylation, and is correlated with immune cell infiltration and enhanced proliferation of pancreatic cancer. Finally, we demonstrate that knockdown of NSD3 alters H3K36me2 methylation, downstream gene expression and EGFR/ERK signaling in pancreatic cancer cells.

**Conclusions:**

We find that expression levels, the presence of genetic variants of NSD family genes, as well as their promoter methylation are correlated with clinical outcomes in cancer, including pancreatic cancer. Our in vitro experiments suggest that NSD3 may be relevant to gene expression regulation and growth factor signaling in pancreatic cancer.

## Graphical abstract

Using multiple databases, we performed gene expression analysis, prognostic analysis, variant analysis, immune analysis, and methylation analysis of the NSD family and identified that NSD3 was highly expressed in pancreatic cancer and associated with poor prognosis, which was validated in our pancreatic cancer cohort. Furthermore, we explored the function and mechanism of NSD3 in pancreatic cancer by in vitro cell experiments. We found that NSD3 may promote the progression of pancreatic cancer by promoting H3K36m2 formation and regulating proliferation‐related gene expression.

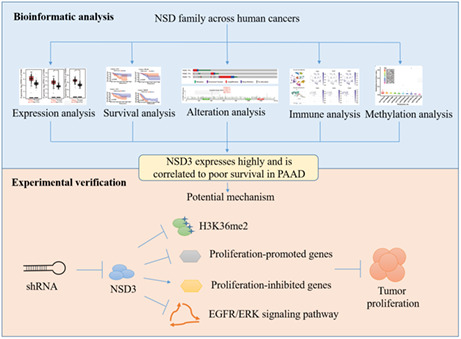

## 1 INTRODUCTION

Cancer is the leading cause of death around the worldwide. In 2020, it was estimated that over 19.3 million people received a cancer diagnosis, and more than 10.0 million people had died from cancer.^1^ Pancreatic cancer is the seventh leading cause of death worldwide, and this is predicted to rise to the second leading cause by 2030.^1^, ^2^ Yet, patients are not diagnosed with pancreatic cancer until they are in an advanced stage, beyond effective surgical and chemotherapeutic intervention.^3^ A better understanding of the mechanisms for, and the underlying causes of pancreatic cancer is essential to improve early detection of this debilitating form of cancer, as well as to discover more effective treatments that can improve clinical outcomes.

It is widely recognized that mutations to essential genes can lead to cancer. Ostensibly, mutations to genes essential to cellular homeostasis, differentiation, and signal transduction are all implicated in cancer. Members of the nuclear receptor‐binding SET domain (NSD) family, including NSD1, NSD2 (WHSC1/MMSET), and NSD3 (Wolf–Hirschhorn syndrome candidate 1‐like 1, WHSC1L1), all play crucial roles in regulating chromatin integrity and histone‐related genes expression by methylating histone H3 on lysine 36 (H3K36) so as to mediate homeostatic gene expression and regulate cellular growth, differentiation, and quiescence.^4^, ^5^, ^6^ As such, it is unsurprising that it has also been reported that alterations and chromosomal translocation of NSD genes are associated with cancer. Of the family members, NSD1 is recognized as the largest gene, located on chromosome 5q35. Aberrant expression of NSD1 is closely related to Sotos syndrome and tumorigenesis, including prostate cancer,^7^ melanoma,^8^ and acute myeloid leukemia.^9^, ^10^ On the contrary, NSD2 encodes a protein paralog of the lowest molecular weight, and its functions are implicated in cancers including multiple myeloma,^11^, ^12^ endometrial cancer,^13^ hepatocellular carcinoma,^14^ and neuroblastoma.^15^ In addition, the NSD3 gene is composed of three main isoforms (NSD3 long/NSD3L, NSD3 short/NSD3s, and WHSC1‐like 1 isoform 9 with methyltransferase activity to lysine/ WHISTLE) and encodes a protein with chromatin‐binding motifs PHD5‐C5HCH, a feature which differs from NSD1 and NSD2 in recognizing histone H3.^16^, ^17^ Its aberrant expression is associated with malignancies including lung cancer,^18^, ^19^ breast cancer,^18^, ^20^ and bladder carcinoma.^21^

Despite evidence for all three NSD family members in cancers, their roles in pancreatic cancer remain poorly characterized. To address this, we analyzed the clinicopathologic features for a series of cancers and explore the association of such features with NSD genes, especially for pancreatic cancer. We also investigated the putative oncogenic role for NSD3 and its potential mechanistic actions in pancreatic cancer cell homeostasis.

## 2 MATERIALS AND METHODS

### 2.1 Gene expression analysis

The “General‐Quick Search” module of GEPIA2 website (http://gepia2.cancer‐pku.cn)^22^ was utilized to study the mRNA expression profiles across cancer and paracancer tissues documented in TCGA and GTEx datasets. The “Expression Analysis‐Box Plots” module of the GEPIA2 website was employed to conduct box plots of mRNA expression profiles for NSD gene family members in tumor and corresponding paracancer tissue samples in PAAD. Log2FC (fold change) > 1 and *p* < 0.01 were deemed significant. “Match TCGA normal and GTEx data” option was selected. The UALCAN portal (http://ualcan.path.uab.edu/analysis.html)^23^ was applied to analyze protein expression profiles of NSD family members from the CPTAC datasets.

### 2.2 Analysis of clinical features

The UCSC Xena (https://xena.ucsc.edu/) database contains clinicopathologic and RNA‐seq data from the TCGA repository and was used to investigate correlations with NSD family members. Gene expression datasets were extracted with Strawberry Perl (version 5.32.1). The correlation between expression profiles for NSD family members, tumor stage, and patient age was determined using the R packages “limma” and “ggpubr.” Significance thresholds for clinical trait associations were set at *p* < 0.05.

### 2.3 Survival analysis

The potential correlation between expression levels for NSD family members and overall survival (OS) and progression‐free survival (PFS), calculated using the Kaplan–Meier method with the log‐rank test and Cox analysis, was explored. The R packages “survival” and “survminer” were utilized to draw survival curves.

### 2.4 Genetic alteration analysis

The characteristics of genetic mutations to NSD genes, including dosage alterations, type of mutation, as well as the relationship between copy number variation and mRNA expression levels for candidate genes in aneuploid regions were estimated using the website tools published in cBioPortal (https://www.cbioportal.org).^24^ Data for OS, disease‐free survival (DFS), disease‐specific survival (DSS), and PFS for cancers with or without NSD3 mutations were gathered. Log‐rank tests were conducted, and Kaplan–Meier curves were prepared to analyze the data. UCSC Xena was employed to estimate somatic mutation data from the TCGA cohort. Spearman's rank was used to perform the correlation of the expression of NSD genes with tumor mutation burden (TMB) and microsatellite instability (MSI) reported in this study. The results were presented as radar maps drawn by the R package “fmsb.”

### 2.5 Immune infiltration analysis

The degree of immune and stromal infiltration across cancers was explored by estimating stromal scores and immune scores using Spearman correlation.^25^ Next, the “Gene” module of TIMER database (https://cistrome.shinyapps.io/timer/)^26^ was applied to examine potential relationships between NSD genes with six immune cell types in PAAD. Tumor Immune Single‐cell Hub (TISCH, http://tisch.comp‐genomics.org)^27^ was applied to evaluate and visualize the expression level of NSD genes across subgroups of immune‐related cells documented in PAAD datasets.

### 2.6 Correlation of NSD3 expression with DNA methylation

Human 450 methylation data from a PAAD cohort described in UCSC Xena were downloaded for analysis. The relationships between NSD genes and methylation‐associated genes were estimated. The correlation of NSD3 methylation with clinical prognosis was evaluated using Kaplan–Meier survival analysis, including OS and PFS (*p* < 0.05 as significant threshold).

### 2.7 PAAD clinical samples

We collected 63 formalin‐fixed, paraffin‐embedded cancer specimens from pancreatic cancer patients who underwent surgical excision between 2014 and 2018 at West China Hospital, Sichuan University (Chengdu, China). Clinical characteristics including age, gender, TNM stage (according to the eighth edition of 2018 National Comprehensive Cancer Network staging criteria), tumor grade, nerve invasion, preoperative CA199, and CEA values were all collected. In total, the patients in our cohort comprises 39 males and 24 females with a mean age of 61.4 years, ranging from 39 to 79 years. Within the cohort, 1 case was well differentiated, 20 cases were moderately differentiated, and 42 cases were poorly differentiated. One patient was classified as stage I, 55 were classified as stage II, 1 was classified as stage III, and 6 were classified as stage IV. Follow‐up data containing survival time and performance status were collected until September 2019. The median overall survival time of these 63 patients was 4 months, ranging from minimum 1 month to maximum 41 months. In addition to this cohort, we collected six paired frozen pancreatic cancer and normal tissues from West China Hospital, Sichuan university. Patients were diagnosed clinically and pathologically with pancreatic ductal adenocarcinoma. None of these patients had been treated with radiotherapy or/and chemotherapy before surgery. This was approved by the Institutional Ethics Committee of West China Hospital, Sichuan university, with authorized informed consent secured from all patients.

### 2.8 Immunohistochemistry

Fixed tissue samples were subjected to paraffin embedding, sectioning, deparaffinating, rehydrating, and antigen retrieval. Following this, sections were incubated with 3% hydrogen peroxide solution for 10 min at room temperature. Slides were then incubated with NSD3 polyclonal antibody (Proteintech) at a dilution of 1:150 overnight at 4°C. The next day, goat anti‐rabbit antibody (Jackson) at a dilution of 1:250 was added to slides and incubated for 40 min at room temperature. Signalstain DAB substrate kit (Cell Signaling Technology) was used to for colorimetric detection. The stained sections were analyzed using a microscope (Pannoramic MIDI). The intensity of staining was classified into four‐categories: 0, no staining; 1, weak; 2, moderate; and 3, strong. The proportion of staining was scored as follows: 0, 0%–5%; 1, 6%–25%; 2, 26%–50%; and 3, >50%. The results of intensity x proportion of each slide were calculated, and the product >3 was considered as high NSD3 expression, and product ≤3 was considered as low NSD3 immunostained signal.

### 2.9 Cell culture

As previously described, AsPc‐1, BxPC‐3, Capan‐1, CFPAC‐1, MIA PaCa‐2, PANC‐1, and hTERT‐HPNE cell lines were obtained from the Cell Bank of the Shanghai Institute of Cells, Chinese Academy of Science (Shanghai, China).^28^ Cells were cultured in Dulbecco Modified Eagle Medium (HyClone) or 1640 medium (HyClone) or IMDM medium (Gibco) supplemented with 10% fetal bovine serum (Excell), 100 U/mL penicillin G, and 100 mg/mL streptomycin (Beyotime) at 37°C in a humidified 5% CO_2_ incubator.

### 2.10 RNA extraction, reverse transcription, and quantitative polymerase chain reaction

RNA was isolated from cell lines and frozen tissues using trizol reagent (Invitrogen, Thermofisher) according to the manufacturer's instructions. Reverse transcription was performed using a PrimeScript™ RT Master Mix (TaKaRa). NovoStart® SYBR qPCR SuperMix Plus (Novoprotein) was applied to conduct real‐time quantitative PCR. The amplification procedure was set as follows: an initial denaturation at 95°C for 30 s, followed by 40 cycles at 95°C for 5 s, 60°C for 30 s, and 72°C for 30 s. The related target gene expression was normalized against β‐actin by relative quantification (2−△△Ct) method. Primers used were listed in Table S1.

### 2.11 Western blotting

Cells were collected and digested in RIPA buffer in the presence of 1% protease inhibitor cocktail (Bimake). Proteins were separated by SDS‐PAGE and then transferred to PVDF membranes (Millipore, USA). After blocking in 5% skimmed milk, the membranes were incubated with antibodies to NSD3 (Cell Signaling Technology), β‐actin (Zhong Shan‐Golden Bridge), Histone‐H3 (proteintech), DiMethyl‐Histone H3 (ZENBIO), EGFR (ZENBIO), phospho‐EGFR (Tyr1173, ZENBIO), ERK1/2 (HUABIO), and phospho‐ERK1/2 (HUABIO) at 4°C overnight. The next day, blots were rinsed before application of a secondary antibody at room temperature for 90 min. Immunocomplexes were detected by electrochemiluminescence reagent (Millipore), with β‐actin as loading control.

### 2.12 Cloning, lentiviral transduction, and generation of stable cell lines

To establish stable *NSD3*‐knockdown cell lines, pre‐designed human NSD3 shRNAs cloned into pLKO.1 lentiviral vector were obtained. For the generation of lentiviral particles, the lentiviral vector and packaging DNA were co‐transfected into 293T cells for 48–72 h, and the medium containing lentiviruses were harvest and transduced to target cells. The shRNA sequences are as follows (shRNA1: 5′‐GGAAGTGTCCACTGGTGTTAA‐3′; shRNA2: 5′‐GGAAGTGTCCACTGGTGTTAA‐3′; shRNA3: 5′‐GCATGTTAGTATCCTCCTACA‐3′).

### 2.13 CCK8 assay, clone formation assay, and EdU incorporation assay

Briefly, cells (2 × 10^3^/well) were seeded onto 96‐well plates and incubated for 0, 1, 2, 3, and 4 days. Ten microliters of CCK8 reagent (TargetMol) per well was added and incubated in 37°C for 2 h. Absorbance was measured at 450 nm under a multifunctional enzyme marker. For clone formation assays, transfected cells (1 × 10^3^/well) were cultured in 6‐well plates for 10–14 days. Cells were fixed by application of 4% formaldehyde in PBS and stained with crystal violet. Assays to label DNA incorporation were carried out using a Cell‐Light Edu Apollo488 in Vitro Kit (RiboBio) according to manufacturer's instructions. Briefly, cells in 24‐well format were incubated with EdU solution for 2 h at 37°C. After fixation in 4% paraformaldehyde, neutralization with glycine solution and permeabilization with 0.5% Triton X‐100, the cells were incubated with Apollo solution for 30 min in the dark, followed which Hoechst 33342 staining was performed. The stained cells were then analyzed using an inverted fluorescence microscope (Nikon).

### 2.14 Differential gene expression analysis and GSEA analysis in a TCGA‐PAAD dataset

RNA‐seq data from TCGA_PAAD (https://portal.gdc.cancer.gov/) cohorts were downloaded and applied to identify differential expression genes (DEGs) between NSD3 high and low expression subgroups. DEGs were extracted by R package “limma.” Gene set enrichment analysis (GSEA) provided by the JAVA program (Version 4.1.0) was used to investigate the downstream‐related pathways affected by differential expression of NSD3 gene. R packages “plyr,” “ggplot2,” “grid,” and “gridExtra” were used to conduct multiGSEA. Abbreviations used in this paper are listed in Table S2.2.

### 2.15 Statistical analysis

R 4.0.5 (https://www.R‐project.org/), GraphPad Prism 5 and SPSS Statistics 26 were used for statistical analysis. Each experiment was repeated at least three times. Data are represented as mean ± SD, and Students' *t*‐tests were applied for calculating *P* values. For studies of clinicopathologic parameters and for IHC signal quantification, chi‐square tests and Fisher's exact tests were applied to detect statistically significant associations with NSD3 expression. The Kaplan–Meier method was used to draw survival curves, and *p* values were calculated using log‐rank test. Cox proportional hazards models were applied to conduct univariate and multivariate survival analyses. *p < 0.05, **p < 0.01, ***p < 0.001.

## 3 RESULTS

### 3.1 Differential expression of NSD genes across human cancers

**FIGURE 1:**
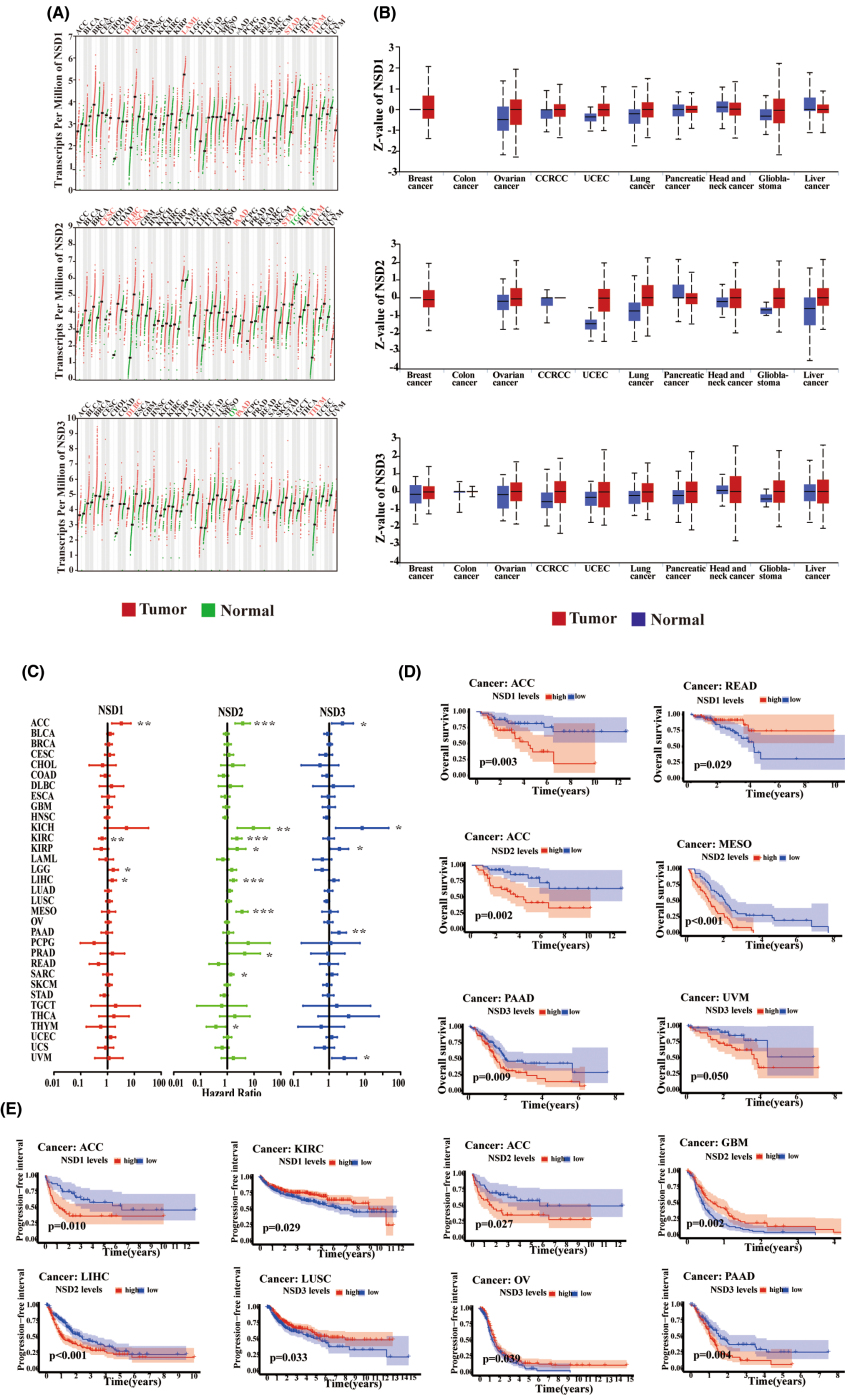
NSD family members are differentially expressed in various cancerous and corresponding normal tissues as well as affecting the prognosis of cancer patients. (A) The mRNA expression level of NSD family in tumor and normal samples across human cancer. (B) The protein expression level of NSD family in tumor and normal samples across human cancer. CCRCC, Clear cell renal cell carcinoma; UCEC, uterine corpus endometrial carcinoma. (C) Forest plots with the hazard ratios and 95% confidence intervals for overall survival in different tumors by Univariate Cox proportional hazard regression models. (D) Correlation between overall survival (OS) and NSD genes from TCGA dataset by Kaplan–Meier method and log‐rank test. (E) Correlation between progression‐free survival (PFS) and NSD genes from TCGA dataset by Kaplan–Meier method and log‐rank test. The TPM value and *Z*‐value were used to display the relative expression of NSD genes. *p < 0.05, **p < 0.01, ***p < 0.001.

We analyzed the mRNA and protein expression levels for NSD family members in cases of cancer documented in TCGA cohorts, GTEx datasets, and CPTAC datasets. Figure 1A shows that mRNA levels for NSD1 are prominent in DLBC, LAML, STAD, and THYM. In contrast, levels of NSD2 mRNA transcripts are prominent in CESC, DLBC, ESCA, PAAD, STAD, and THYM, and weak in TGCT. Furthermore, NSD3 mRNA expression levels are high in DLBC, PAAD, and THYM, but low in OV. Protein expression of NSD family members across cancers is also widespread (Figure 1B). Next, we explored the differential expression of NSD genes according to tumor stage and patient age. As shown, we found that NSD genes were differentially expressed in different stages (Figure S1). For instance, NSD1 expression was significantly different between stage I and stage IV ACC cases (*p* = 0.0044), KIRC cases (*p* = 0.03), and THCA cases (*p* = 0.0035). On the contrary, significant differences in NSD2 expression were observed for samples of stage I and stage IV ACC samples (*p* = 0.0013), COAD samples (*p* = 0.00094), ESCA samples (*p* = 0.018), and SKCM samples (*p* = 0.0047). Furthermore, NSD3 expression was significantly different between stage I and stage II samples for COAD (*p* = 0.0031), LUAD (*p* = 0.0023), PAAD (*p* = 0.044), SKCM (*p* = 0.014), and STAD (*p* = 0.027). In terms of potential differences in age, patients aged <65 years in BRCA (*p* = 0.042), ESCA (*p* = 0.022), LIHC (*p* = 0.033), and PAAD (*p* = 0.009) were correlated with high NSD1 expression, while patients aged <65 years in ESCA (*p* = 0.018), HNSC (*p* = 0.039), LAML (*p* = 0.0079), and LIHC (*p* = 0.002) were correlated with high NSD2 expression, while levels of NSD3 were significantly elevated in patients aged <65 years with ESCA (*p* = 0.021), BLCA (*p* = 0.026), KIRC (*p* = 0.015), and LIHC (p < 0.001) (Figure S2). Thus, NSD gene expression is correlated with human cancers in different ways, both in clinical stage and in patient age.

### 3.2 Expression levels for NSD genes are associated with patient survival

We next explored putative links between expression levels for NSDs and patient survival. Using the Cox proportional hazards model analysis approach (Figure 1C), we found that NSD1 expression is significantly correlated in ACC, LGG, and LIHC, while it is anti‐correlated in KIRC cases. In the case of NSD2 expression, levels are significantly correlated for poor survival in ACC, KICH, KIRC, KIRP, LIHC, MESO, and PRAD. Furthermore, elevated NSD3 expression was correlated with poor survival for ACC, KICH, KIRP, PAAD, and UVM. We also performed Kaplan–Meier survival analysis of OS to find that elevated NSD1 expression is correlated with a poorer survival in ACC, and improved patient outcomes in READ and KIRC (Figure 1D and Figure S3A). Patients with low NSD2 expression in ACC, MESO, and LIHC, and high expression profiles of NSD2 in HNSC and THYM, were correlated with improved OS. Also, elevated expression levels for NSD3 are associated with poorer survival for KICH, PAAD, and UVM, as well as prolonged OS of LUSC. In the case of PFS, patients with low expression levels for NSD1 in ACC and high expression in KIRC are associated with a longer PFS, while patients with high levels of NSD2 in ACC, LIHC, LUAD, MESO, and PRAD, as well as low NSD2 expression in GBM have a poor PFS (Figure 1E and Figure S3B). Furthermore, prolonged PFS is correlated with low NSD3 expression levels in PAAD, and high expression of NSD3 in LUSC and OV. Thus, these results demonstrate that the expression levels for NSD genes are significantly correlated with clinical severities, overall survival, and tumor progression.

### 3.3 NSD genetic traits, immune infiltration analysis in the TGCA cancer cohort

**FIGURE 2:**
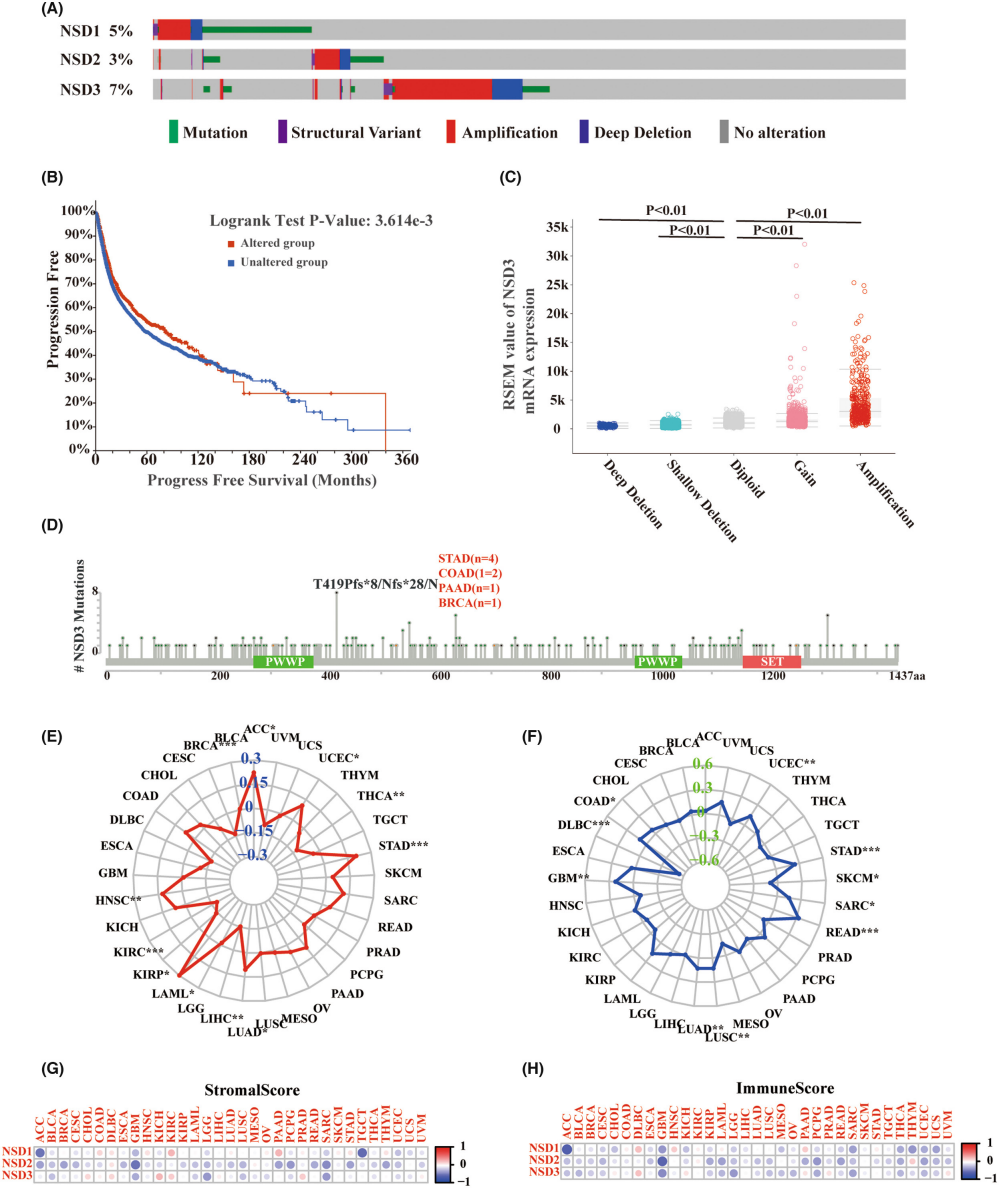
Alteration feature and immune analysis of NSD genes across tumors. (A) the alteration frequency of NSD genes in pan‐cancer. (B) The potential correlation between NSD3 alteration and progression‐free survival of pan‐cancer. (C) The relationship between mRNA of NSD3 and copy number alteration, including deep deletion (*n* = 114), shallow deletion (*n* = 2125), diploid (*n* = 5226), gain (*n* = 2018), and amplification (*n* = 406). RSEM was used to show the mRNA expression level of NSD3. RSEM: Batch normalized from Illumina HiSeq_RNASeqV2. (D) The detailed mutation sites of NSD3 across tumors. (E) Association between NSD3 expression and tumor mutational burden (TMB) in pan‐cancer. (F) Association between NSD3 expression and microsatellite instability (MSI) across human cancers. Correlation matrix plots to show the correlation of NSD genes expression with stromal scores (G) and immune scores (H) across cancers based on ESTIMATE algorithm by spearman correlation test. Size of the dots means the absolute value of the correlation coefficients. *p < 0.05, **p < 0.01, ***p < 0.001.

We analyzed the genetic characteristics of NSD genes in pan‐cancer patients documented in the TCGA‐PAAD database. As shown, the frequencies of genetic alterations in NSD1, NSD2, and NSD3 across cancers were 5%, 3%, and 7%, respectively (Figure 2A). In the case of NSD1 and NSD2 in TCGA cohorts, “mutation” was the primary alteration type, while “amplification” is the primary alteration described for NSD3. We next focused on NSD3 as it was the most frequently alteration gene among family members.

The presence of NSD3 mutations was negatively correlated with improved outcomes in PFS (*p* = 0.0036), but not DSS (*p* = 0.115), DFS (*p* = 0.349), or overall survival (*p* = 0.798) (Figure 2B and Figure S4). Furthermore, amplification and copy number gain of NSD3 were associated with high mRNA levels, while deletions correlate with decreased transcript levels (Figure 2C). In terms of mutations to the gene locus, we found a preponderance of missense NSD3 mutations. Also, recurrent T419Pfs*8/Nfs*28/N mutations were detected in eight cases, and consisting of four cases of STAD, two cases of COAD, as well as single cases of BRCA and PAAD. In addition, when we examined NSD3 expression relative to TMB, we found that expression levels were negatively correlated with TMB for THCA, LIHC, KIRC, and BRAC, while a significant association was found for ACC, UCEC, STAD, LUAD, LAML, and HNSC (Figure 2E). Furthermore, we found that MSI for UCEC, STAD, SARC, READ, LUSC, LUAD, GBM, and COAD were positively correlated with NSD3 expression, while a negative correlation of expression levels was detected for MSI in SKCM and DLBC (Figure 2F).

To explore potential association between NSD expression and infiltrating stromal cells and immune cells in cancer, we applied the ESTIMATE method to detect statistically significant observations. As shown in Figure 2G, patients with low NSD1 expression were significantly correlated with decreased stromal score in ACC and TGCT, while low NSD2 expression was linked to stromal score in GBM and SARC. Furthermore, NSD3 expression is associated with stromal score in LGG and SARC. In addition, immune scores are adversely correlated with ACC and GBM, and NSD2 and NSD3 are negatively correlated with immune scores for GBM.

### 3.4 Bioinformatic analysis of NSD genes in pancreatic cancer

**FIGURE 3:**
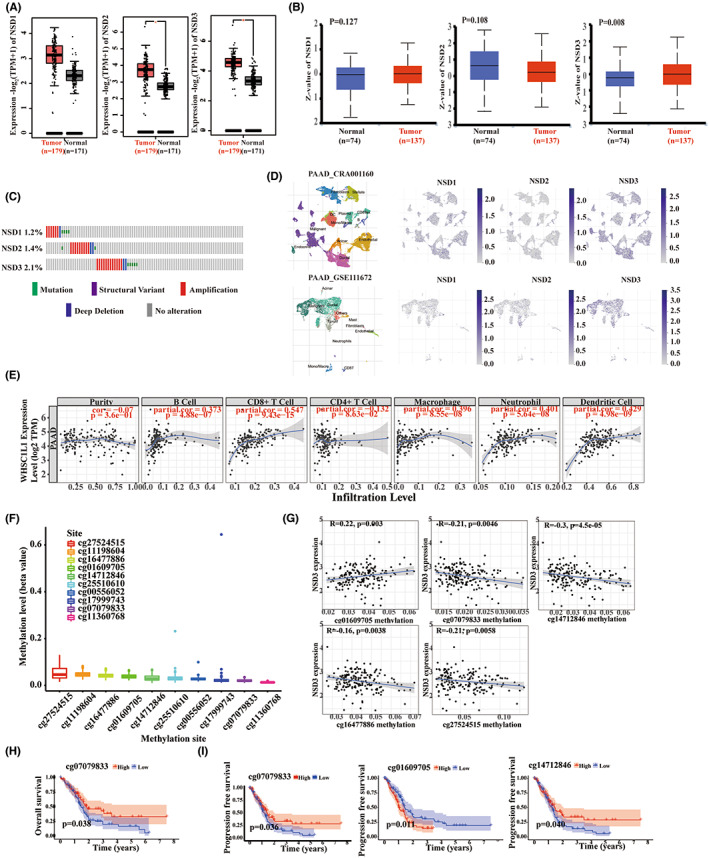
Bioinformatic analysis of NSD family in pancreatic cancer. (A) The mRNA expression level of NSD family in tumor and normal samples in PAAD. (B) The protein expression level of NSD family in tumor and normal samples in PAAD. (C) the alteration frequency of NSD genes in PAAD. (D) The expression visualization of NSD family in different cell types across PAAD datasets, including PAAD_CRA001160 and PAAD_GSE111672, using TISCH database. (E) The correlation between NSD3 and immune infiltrating cells in PAAD using TIMER. Log2 TPM was used to show the NSD3 (WHSC1L1) expression level. TPM: transcripts per million. (F) The methylation levels of 10 methylation sites in NSD3 promoter region. (G) The relationship between methylation levels of 5 methylation sites and NSD3 expression. (H) The Kaplan–Meier survival curves of overall survival (OS) with methylation levels of cg07079833 methylation site using log‐rank test. (I) The Kaplan–Meier survival curves of progression‐free survival (PFS) with methylation levels of 3 methylation sites using log‐rank test.

We next focused on NSD genes in pancreatic cancer. As shown, the mRNA expression levels for NSD2 and NSD3 are significantly elevated in tumor samples compared to normal samples, while quantification of protein levels reveal that NSD3 is upregulated in tumor samples (Figure 3A,B). We used GEPIA2 web to explore the relationship between NSD genes and PAAD prognosis and found that NSD3 is related to OS and DFS of PAAD (Figure S3C,D). Moreover, the genetic alteration frequencies for NSD1, NSD2, and NSD3 in the PAAD cohort were 1.2%, 1.4%, and 2.1%, respectively (Figure 3C), with “amplification” as the primary alteration type and NSD3 identified as the most frequently altered gene. When we utilized the TISCH platform to estimate NSD gene expression in PAAD_CRA001160 and PAAD_GSE111672 cohort (Figure 3D and Figure S5), we found that NSD1 was predominantly enriched in the B‐cell subset in PAAD_CRA001160 cohort as well as the mast subset in PAAD_GSE111672 cohort. In contrast, NSD2 was enriched in proliferative T cells (Tprolif) in PAAD_GSE111672 cohort and B‐cell subset in PAAD_CRA001160 cohort. Furthermore, NSD3 is enriched in CD8 T cells and malignant subsets in the PAAD_GSE111672 cohort.

We focused on NSD3 for additional investigations in pancreatic cancers as we found that, compared to NSD1 and − 2, it was the most prominently expressed gene across NSD members, the most frequently altered NSD gene, and that it was also significantly correlated with cancer prognosis as well as immune infiltration in PAAD. We began by studying potential relationships between NSD3 expression and B cells, CD8^+^ T cells, CD4^+^ T cells, macrophages, neutrophils, and dendritic cells in PAAD (Figure 3E). As shown, NSD3 expression was positively correlated with B cells, CD8+T cells, macrophages, neutrophils, and dendritic cells infiltration, but was not relevant to CD4^+^ T cells.

In addition to NSD3 expression in PAAD immune cell types, we also analyzed promoter methylation sites for this gene, particularly mapping to 10 distinct sites (Figure 3F), including cg27524515, cg11198604, cg16477886, cg01609705, cg14712846, cg25510610, cg00556052, cg17999743, cg07079833, and cg11360768. Methylation at these NSD3 sites were generally low, with cg27524515 identified as the most prominent, yet NSD3 expression is independent of promoter methylation except for cg01609705, cg07079833, cg14712846, cg16477886, and cg27524515 (Figure 3G and Figure S6A). Furthermore, high methylation levels for cg07079833 (*p* = 0.038) were linked to prolonged OS (Figure 3H), while patients with low methylation level of cg01609705 and high methylation level of cg14712846 (*p* = 0.040) or cg07079833 (*p* = 0.036) had prolonged PFS (Figure 3I). Levels of methylation for other sites were not significantly correlated to OS or PFS (Figure S6B,C). Thus, we identified distinct signatures for site‐specific methylation of NSD3 genomic loci that were correlated with clinical states of patients with pancreatic cancer.

### 3.5 Evaluating the prognostic value and expression patterns for NSD3 in PAAD patients and in cell lines

**FIGURE 4:**
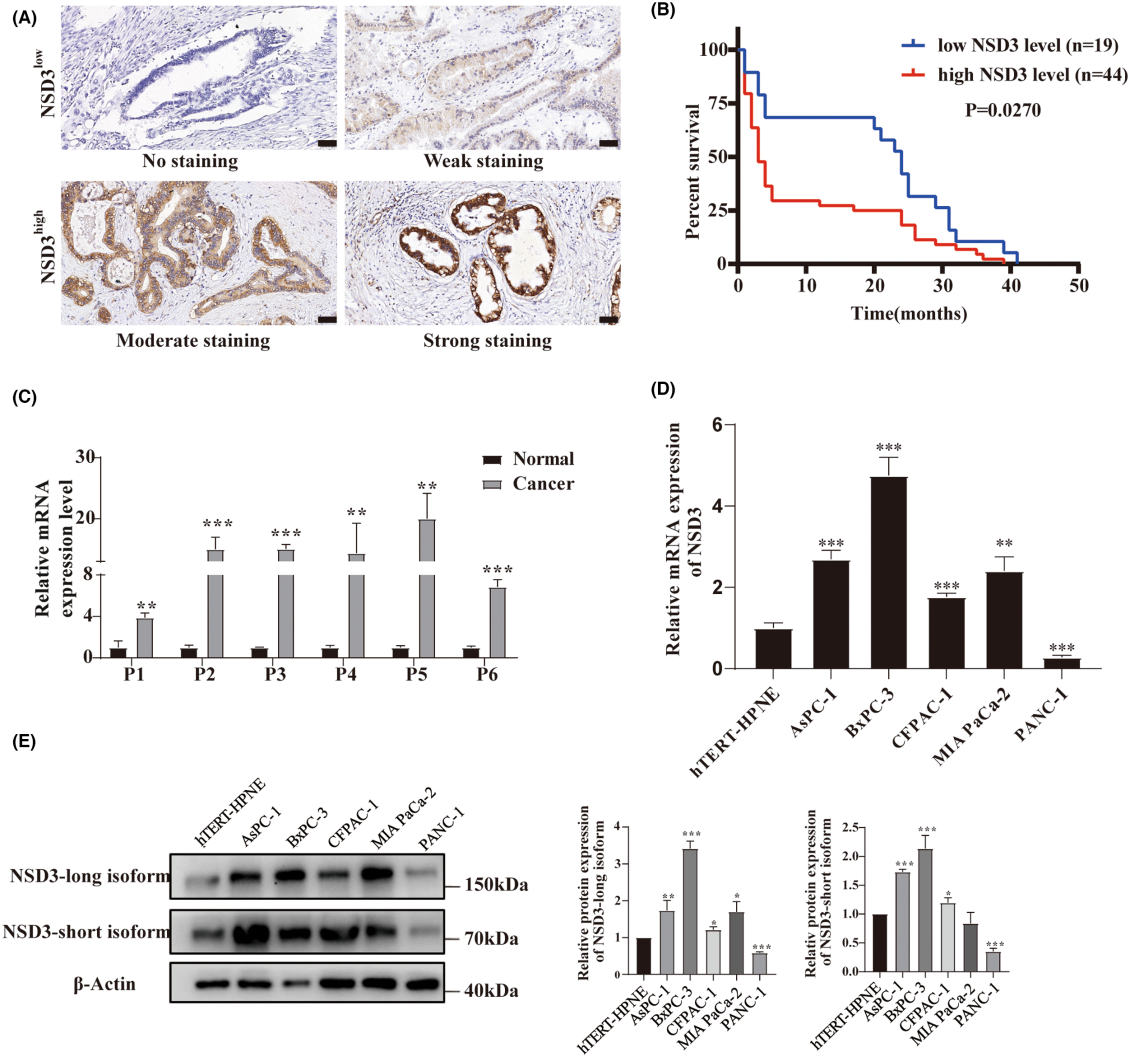
Expression pattern of NSD3 in pancreatic cancer. (A) Representative immunohistochemical staining images of low NSD3 expression (no staining and weak staining) and high NSD3 expression (moderate and and strong staining) in pancreatic ductal adenocarcinoma tissues, respectively (original magnification ×400). (B) Kaplan–Meier survival curves of patients with pancreatic ductal adenocarcinoma (*n* = 63). (C) Relative NSD3 mRNA expression level between tumor and paired normal tissues from 6 patients with pancreatic ductal adenocarcinoma. P: patient. (D) Relative mRNA expression of NSD3 in pancreatic cancer cell lines and a normal pancreatic ductal cell line. (E) Protein expression of NSD3 in pancreatic cancer cell lines and a normal pancreatic ductal cell line. *p < 0.05, **p < 0.01, ***p < 0.001.

To evaluate the prognostic potential for *NSD3* expression in PAAD, we analyzed clinical specimens by IHC and found that NSD3 immunostained signals in PAAD tissue were markedly different between samples (Figure 4A). Furthermore, KM analysis revealed that PAAD patients with a higher expression levels for NSD3 were associated with poor overall survival (Figure 4B). Next, we sought to externally validate our findings for NSD3 in TCGA cohort data by analyzing NSD3 expression profiles and clinicopathologic features in an independent collection of pancreatic cancer samples (Table 1). As shown, of 63 PAAD specimens, 44 (69.8%) were consider as high NSD3 expression, and 19 (30.2%) were consider as low NSD3 expression. Statistical analysis shows that NSD expression is independent of age (*p* = 0.518), sex (*p* = 0.484), TNM stage (*p* = 0.664), tumor grade (*p* = 0.120), nerve invasion (*p* = 0.212), preoperative CA199 value (*p* = 0.311), and preoperative CEA value (*p* = 0.115). In Table 2, univariate analysis with the Cox proportional hazards model identifies high NSD3 expression (*p* = 0.006), advanced TNM stage (*p* = 0.011), and tumor grade of poor differentiation (*p* = 0.001) as statistically significant risk factors influencing the clinical survival of PAAD patients. Finally, we performed multivariate analysis and discovered that both NSD3 expression (HR = 2.917, *p* = 0.018) and tumor grade (HR = 3.778, *p* = 0.005) were informative as prognostic markers for clinical outcome in patients (Table 3). Therefore, NSD3 expression levels may be a potential prognosticator of PAAD clinical outcomes.

**TABLE 1:** Association of NSD3 expression and clinicopathologic parameters.

Parameters	*n*	Low expression	High expression	P value
Number of patients	63	19	44	
Age				
≤60	26	9	17	0.518
>60	37	10	27	
Sex				
Male	39	13	26	0.484
Female	24	6	18	
TNM stage				
I–II	56	18	38	0.664
III–IV	7	1	6	
Grade				
Well and moderate	21	9	12	0.120
Poor	42	10	32	
Nerve invasion				
Yes	47	12	35	0.212
No	16	7	9	
Preoperative CA19‐9 value				
<37	13	2	11	0.311
≥37	50	17	33	
Preoperative CEA value				
<5	47	17	30	0.115
≥5	16	2	14	

**TABLE 2:** Univariate Cox proportional hazards analysis for survival of PDAC patients.

Variable	Hazard ratio	95% confidence interval	*p* value
NSD3 expression(high/low)	3.401	1.421–8.140	0.006*
Age (≤60/>60)	0.820	0.440–1.531	0.534
Gender (female/male)	0.771	0.402–1.477	0.432
TNM stage (III–IV/I–II)	2.807	1.272–6.199	0.011*
Grade (poor/moderate and well)	4.375	1.798–10.648	0.001*
Nerve invasion (no /yes)	1.070	0.522–2.190	0.854
Preoperative CA19‐9 value (≥37/<37)	0.878	0.418–1.845	0.731
Preoperative CEA value (≥5/<5)	1.649	0.835–3.255	0.150

* *p* < 0.05 was considered statistically significant.

**TABLE 3:** Multivariate Cox proportional hazards analysis for survival of PDAC patients.

Variables	Hazard ratio	95.0% CI	*p* value
TNM stage	1.853	0.836–4.107	0.129
Grade	3.778	1.502–9.503	0.005*
NSD3 expression	2.917	1.203–7.074	0.018*

* *p* < 0.05 was considered statistically significant.

We find that tumor tissues express high levels of NSD3 compared to paired normal tissues (Figure 4C). Indeed, levels of NSD3 are markedly different between pancreatic ductal normal cell and hTERT‐HPNE cells. We also characterized a series of pancreatic cancer cell lines (AsPc‐1, BxPC‐3, Capan‐1, CFPAC‐1, MIA PaCa‐2, PANC‐1) to find that NSD3 expression is elevated to different degrees across samples (Figure 4D,E).

### 3.6 Suppression of NSD3 impairs proliferation of pancreatic cancer cell lines and leads to reductions in H3K36 dimethylation and gene expression changes

**FIGURE 5:**
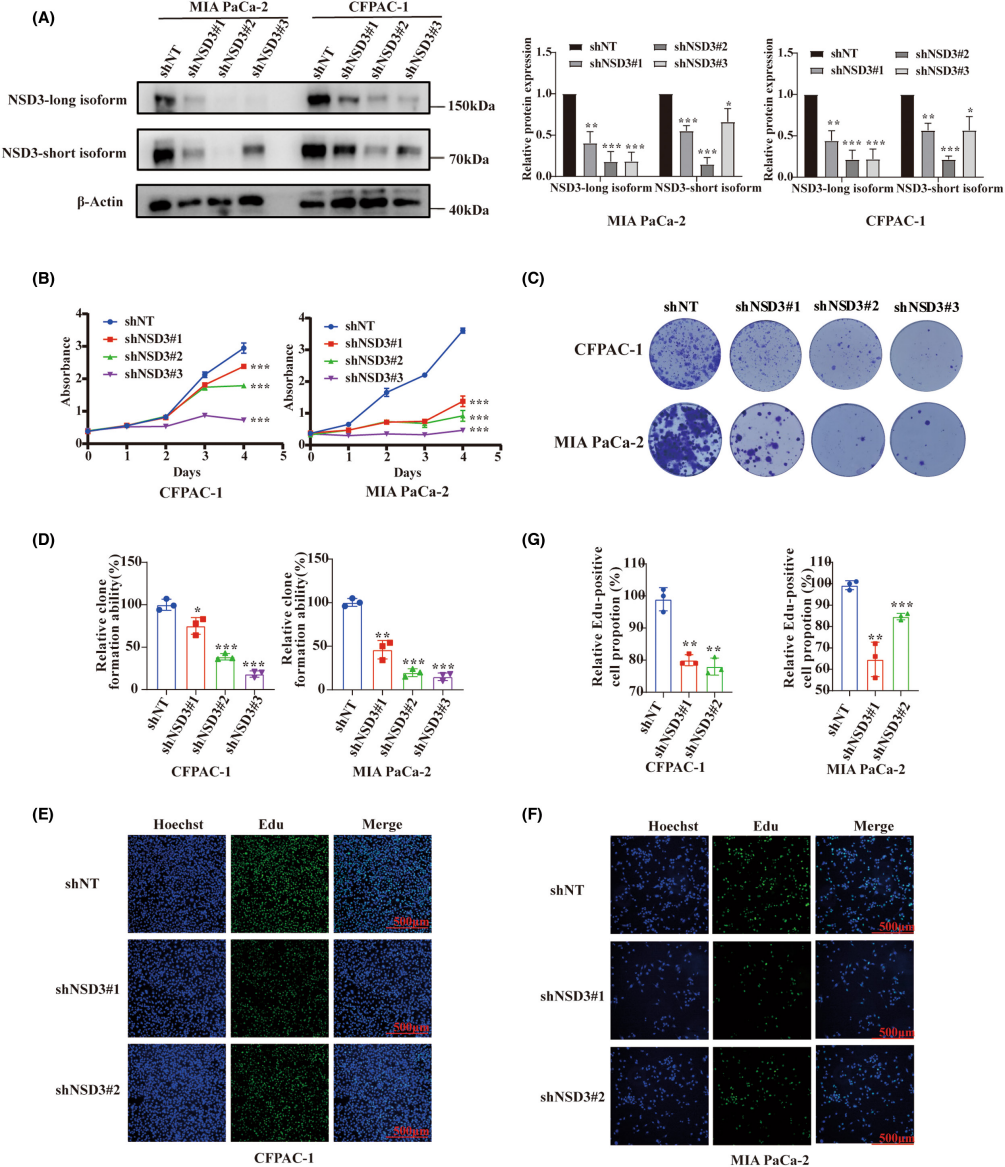
Knockdown NSD3 inhibits proliferation of pancreatic cancer cells. (A) Western blotting result and corresponding graphs presented that NSD3 protein expression level after after shRNA transfection of CFPAC‐1 and MIA PaCa‐2 cells. (B) Cell proliferation of CFPAC‐1 and MIA PaCa‐2 cells after knockdown NSD3 measured by CCK‐8 assays. (C) Cell proliferation of CFPAC‐1 and MIA PaCa‐2 cells after knockdown NSD3 measured by clone formation assays. (D) Statistic analysis of clone formation. Cell proliferation of CFPAC‐1(E) and MIA PaCa‐2 (F) cells after knockdown NSD3 measured by Edu assays. (G) Statistic analysis of Edu assays. *p < 0.05, **p < 0.01, ***p < 0.001.

**FIGURE 6:**
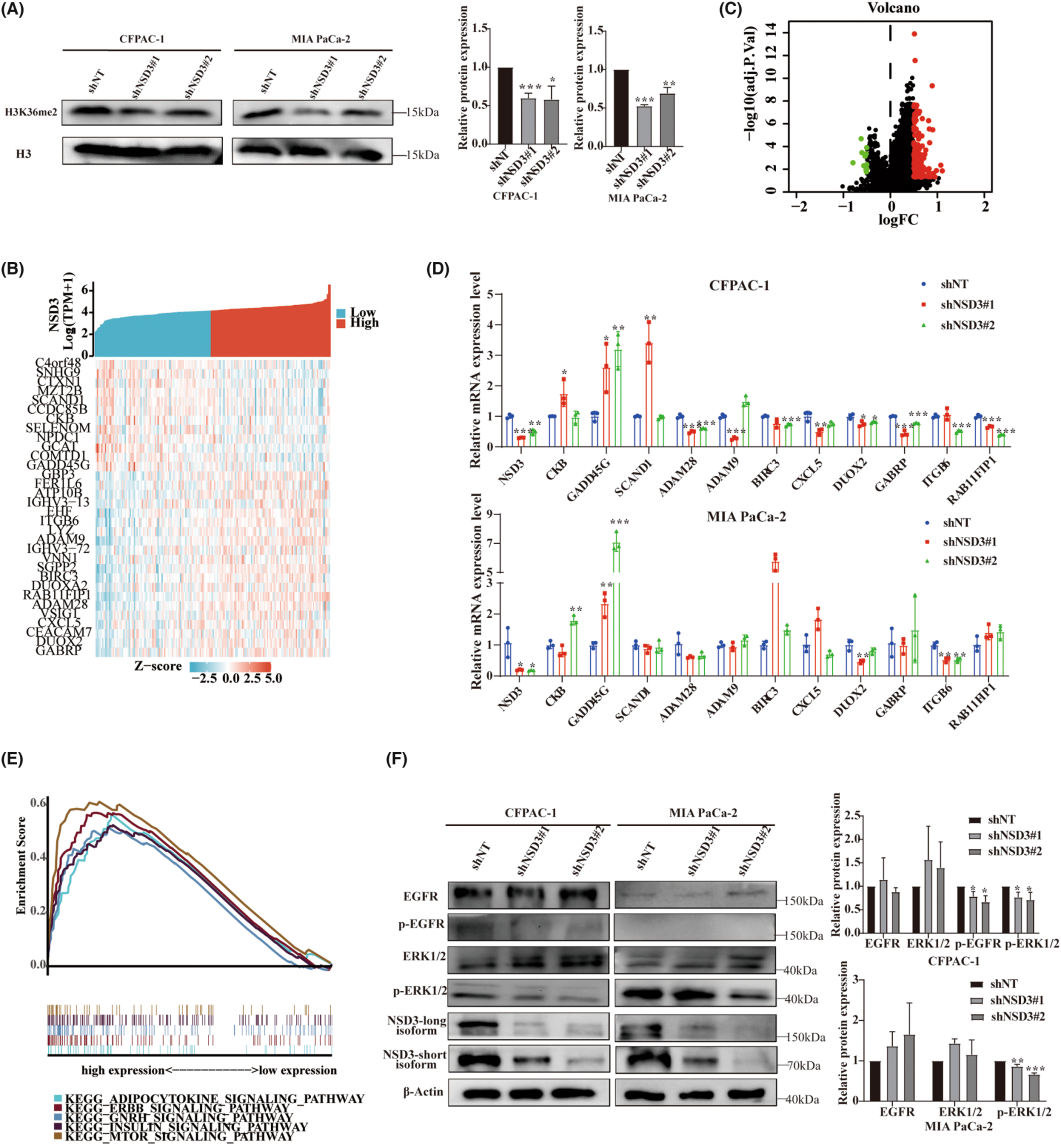
NSD3 induces H3K36me2 in PAAD, regulates the transcription of some potential genes, and affects the EGFR/ERK signaling pathway. (A) Knockdown NSD3 leads to decreased global dimethylated H3K36 (H3K36me2) in pancreatic cancer cells, with Histone H3 as a loading control. (B) Heat map of the top 20 upregulated genes as well as only 12 downregulated genes. (C) Volcano Plot of differentially expressed genes (DEGs) of NSD3 in TCGA‐PAAD cohort. (D) Validation of the selected 11 genes related to tumor proliferation via quantitative real‐time PCR. (E) MultiGSEA analysis of NSD3‐related signaling pathways in PAAD. (F) Effect of NSD3 knockdown on EGFR, p‐EGFR, ERK1/2 and p‐ERK1/2 levels in pancreatic cancer cells assessed by Western blotting, and corresponding graphs of relative protein levels are alongside the blot images.*p < 0.05, **p < 0.01, ***p < 0.001.

We performed a series of studies using MIA PaCa‐2 and CFPAC‐1 cell lines to examine the consequences of NSD3 suppression. As shown, using shRNA against NSD3 (shNSD3), levels of immunoblotted NSD3 protein in these cells were reduced (Figure 5A). Next we carried out a CCK8 assay, colony formation assays, and Edu assays to demonstrate that knockdown of NSD3 inhibits proliferation, colony formation, and DNA incorporation by pancreatic cancer cells (Figure 5B–G). Given that NSD3 is a dimethyltransferase of H3K36, we next surveyed H3K36 dimethylation to find that global H3K36me2 expression is decreased following knockdown NSD3 (Figure 6A). Furthermore, we explored the potential downstream mechanism by which NSD3 gene influences cellular gene expression by identifying significant genes correlated with NSD3 gene expression and pancreatic cancer in TCGA‐PAAD cohorts. As shown, we found total of 148 DEGs (Table S3), the top 20 upregulated genes and only 12 downregulated genes of which included COMTD1 (catechol‐o‐methyltransferase domain containing 1), C4orf48 (chromosome 4 open reading frame 48), CCDC85B (coiled‐coil domain containing 85B), CXCL5 (C‐X‐C motif chemokine ligand 5), and others (Figure 6B,C). We then selected 11 genes related to tumor proliferation to validate their gene expression relationships to NSD3 by Quantitative Real‐time PCR, including CKB (Creatine Kinase B),^29^ GADD45G (growth arrest and DNA damage inducible gamma),^30^ SCAND1 (SCAN Domain Containing 1),^31^ ADAM28 (ADAM metallopeptidase domain 28),^32^ ADAM9 (ADAM metallopeptidase domain 9),^33^ BIRC3 (Baculoviral IAP Repeat Containing 3),^34^ CXCL5 (C‐X‐C motif chemokine ligand 5),^35^ DUOX2 (dual oxidase 2),^36^, ^37^ GABRP (gamma‐aminobutyric acid type A receptor subunit Pi),^38^ ITGB6 (integrin subunit beta 6),^39^ and RAB11FIP1 (RAB11 family interacting protein 1).^40^ As shown, downregulation of NSD3 results in elevated mRNA levels for CKB, GADD45G, and SCAND1, and decreased mRNA expression level of ADAM28, ADAM9, BIRC3, CXCL5, DUOX2, GABRP, ITGB6, and RAB11FIP1 in CFPAC‐1. In MIA PaCa‐2, expression levels for CKB and GADD45G mRNA were upregulated while DUOX2 and ITGB6 levels were reduced following NSD3 knockdown, altogether suggesting that these genes may be influenced by NSD3 expression. We performed GSEA analysis of these DEGs to find enrichment in ERBB signaling, adipocytokine signaling, GNRH signaling, insulin signaling, and mTOR signaling in context with NSD3 expression (Figure 6E). Next, we verified that, following knockdown of NSD3, steady‐state levels of p‐EGFR immunoblotted signal are decreased in CFPAC‐1 cells, while the EGFR signals were not markedly affected (Figure 6F). In contrast, changes to p‐EGFR immunoblotted signals were not different MIA PaCa‐2 following NSD3 knockdown. In addition, we also studied EGF/ERK signaling in both CFPAC‐1 and MIA PaCa‐2 to find that steady‐state levels of p‐ERK are decreased upon NSD3 knockdown, while the expression of ERK1/2 is not significantly reduced. Taken together, these findings suggest NSD3 expression influences the proliferation of pancreatic cancer cells, influences global methylation patterns in the genome of cells, and mediates downstream genes relevant to ERBB and EGFR/ERK signaling.

## 4 DISCUSSION

Genes that encode histone methyltransferase proteins, including members of the NSD family of genes, are relevant to cancer.^41^ Indeed, our study has demonstrated that genetic alterations to NSD genes underlie a variety of cancer subtypes. Within cells, NSD proteins can mono‐ and dimethylate H3K36 to give rise to H3K36me1 and H3K36me2 epigenetic marks.^5^, ^42^ There is increasing evidence that suggests that disruptions to NSD genes or their expression levels lead to dysregulate of H3K36 methylation, leading to developmental abnormalities as well as cancer.^43^, ^44^ In this study, we have systematically analyzed the expression of NSD genes and evaluated the prognostic value for clinical outcomes in cancer.

We find that the expression levels of the NSD genes are correlated with distinct cancer subtypes and clinical severities. For example, we find that prominent NSD1 expression is associated with poor outcomes for DLBC, LAML, STAD, and THYM, while high NSD2 expression is correlated to poor outcomes in CESC, DLBC, ESCA, PAAD, STAD, and THYM, but anti‐correlated with TGCT. Furthermore, elevated NSD3 expression is correlated with poor outcomes for DLBC, PAAD, and THYM. In addition to cancer subtypes, the expression of NSD genes is associated with tumor stage and patient age. To complement these studies, we validated our findings in an independent collection of tissue samples and clinical profiles representing 33 cancer subtypes. Furthermore, we demonstrate that NSD3 is a potential prognosis marker for pancreatic cancer, and this finding is consistent with recent studies showing that elevated NSD3 levels are correlated with poor clinical outcome, recurrence, and metastasis in breast cancer.^45^, ^46^

Furthermore, compared to NSD1 and −2, we find that genetic variation and amplification to NSD3 is most prevalent in cancer. Indeed, *NSD3* is located in chromosome 8p11.2 and has been reported to be amplified in human primary cancers and in cells lines from cancers of the breast, pancreas, and lung.^18^, ^20^, ^47^, ^48^ Additionally, several *NSD3* missense mutations, such as E1181K and T1232A, have been proposed to enhance the growth of cancer cells and xenograft tumors, through disruption of an auto‐inhibitory loop within the NSD3 protein product so as to augment its enzymatic function.^19^, ^49^ Furthermore, genetic fusions of NSD3 with NUP98 and NUTM1 genes have been described in patients with AML as well as myelodysplastic syndrome, and primary pulmonary NUT carcinoma, respectively.^50^, ^51^ Finally, NSD3 expression levels are correlated with TMB and MSI, both representing promising predictive biomarkers of clinical characteristics and prognosis^52^, ^53^; however, no significant correlation between NSD3 expression and TMB as well as MSI in PAAD was detected. Nevertheless, our findings indicate that NSD3 may be informative for delineating clinical prognoses across multiple cancer types.

In cancer, epigenetic regulation is relevant to immune cell functions that mediate anti‐tumor immunity.^54^, ^55^, ^56^ For example, expression of histone‐lysine N‐methyltransferase 2 (KMT2) genes is associated with immune infiltration and modification of the tumor microenvironment (TME).^57^ In the case of NSD genes, Wang and colleagues reported that NSD3 promotes antiviral innate immunity by methylating IRF3,^58^ while Kim and colleagues reported that elevated NSD3 expression is linked to a reduction in CD8^+^ T cells and high CD274 expression.^46^ In our study, we found that NSD genes are correlated with stromal and immune scores in cancer. Moreover, in pancreatic cancer, NSD3 expression is related to infiltration of B cells, CD8^+^ T cells, macrophages, neutrophils, and dendritic cells. We interpret these findings to suggest that NSD3 is relevant to immune cell recruitment in cancer, and future studies would focus on the relevant cell types through which NSD3 mediates its functions in tumors.

In addition to expression levels, we found that methylation levels for NSD3 were low in PAAD. We speculate that this could alleviate negative signaling pathways that block tumor proliferation.Specific to *NSD3*, this gene encodes three isoforms, as follows. The first is a 1437 amino acid polypeptide important for cell growth, cell cycle, and apoptosis.^59^ The NSD3L isoform interacts with EZH2 and RNA polymerase II to influence H3K36me2/3‐dependent transactivation of genes, including those related to NOTCH signaling in breast cancer.^45^ The second, NSD3S, encodes a shorter isoform of 645 amino acids comprising an amino‐terminal PWWP domain, and lacks the catalytic SET domain.^60^, ^61^ NSD3S influences gene expression and genome stability through its interactions with bromodomain‐containing protein 4 (BRD4),^62^, ^63^ and also mediates oncogenesis by suppressing the degradation of MYC protein.^64^ The third isoform, WHISTLE, is composed of 506 amino acids and is less relevant to cancer.^65^, ^66^ NSD3L and NSD3S are co‐expressed in certain tissues,^20^, ^60^ while WHISTLE is expressed primarily in testis and bone marrow mononuclear cells from patients with acute myeloid leukemia or acute lymphoblastic leukemia.^65^ In this study, we have investigated the NSD3L and NSD3S isoforms in pancreatic cancer cell lines to find that steady‐state levels of both are elevated in pancreatic cancer cells. Also, we found that downregulation of NSD3L and NSD3S inhibits proliferation of such cells, and this result is consistent with a recent study.^67^ In addition to protein levels, NSD3 induces global H3K36 dimethylation in pancreatic cancer cells and influences the mRNA levels for genes including ADAM28, ADAM9, BIRC3, CXCL5, DUOX2, GABRP, ITGB6, and RAB11FIP1. Additionally, we find that EGFR/ERK signaling pathway was disrupted by NSD3 knockdown, a finding that is consistent with current findings,^68^, ^69^ as well as a study in head and neck cancer.^70^ Indeed, a study by Yi and colleagues also showed that NSD3 overexpression enhanced the phosphorylation level of ERK1/2 in colorectal cancer, while inhibition of ERK1/2 significantly decreased proliferation.^71^ Taken altogether, we find that NSD3 expression and function is crucial to pancreatic cancer, and such features for NSD3 may be relevant to multiple cell types within the TME.

## 5 CONCLUSION

In conclusion, NSD genes are relevant to a broad spectrum of cancers, and their expression levels are correlated with clinical outcomes in distinct ways. We find that NSD3 expression is strongly associated with clinical prognosis in pancreatic cancer, with genetic amplification of NSD3, low levels of NSD3 promoter methylation and immune cell infiltration to tumor sites as correlated features. We find that knockdown of NSD3 leads to altered H3K36 dimethylation, disruptions to downstream gene expression and impairment in EGFR/ERK signaling. Altogether, we conclude that NSD genes may be informative to prognosticate for cancer. Furthermore, NSD3 may be a potential target for the design of novel pancreatic cancer therapies.

## AUTHOR CONTRIBUTIONS

**Qunli Xiong:** Conceptualization (lead); visualization (lead); writing – original draft (lead); writing – review and editing (lead). **Ying Zhou:** Data curation (equal); visualization (equal). **Su Zhang:** Formal analysis (equal); visualization (equal). **Yaguang Zhang:** Writing – original draft (equal); writing – review and editing (equal); funding acquisition (supporting). **Yongfeng Xu:** Formal analysis (equal); software (equal). **Yang Yang:** Formal analysis (equal); software (equal). **Congya Zhou:** Methodology (equal). **Zhu Zeng:** Writing – original draft (supporting); writing – review and editing (supporting). **Junhong Han:** Funding acquisition (supporting); resources (equal); supervision (equal). **Qing Zhu:** Conceptualization (equal); funding acquisition (lead); resources (equal); supervision (equal).

## CONFLICT OF INTEREST STATEMENT

The authors declare that the research was conducted in the absence of any commercial or financial relationships that could be construed as a potential conflict of interest.

## ETHICAL APPROVAL STATEMENT

All experiments using specimens were approved by the ethics committee and performed strictly abiding by relevant regulations of the Declaration of Helsinki under the prerequisite of obtaining written informed consent.

## Supporting information


Data S1.

Table S1.

## Data Availability

The datasets presented in this study can be found in online repositories. The names of the repository/repositories and accession number(s) can be found in the article/Supplementary Material.
