# Supplementary material for: NSD3, a member of nuclear receptor‐binding SET domain family, is a potential prognostic biomarker for pancreatic cancer
Source: Cancer Med. 2023 Apr 16;12(9):10961–78. doi: 10.1002/cam4.5774 (PMC10225198; doi:10.1002/cam4.5774)
Supplement: Supplementary file 1 — Data S1. [file CAM4-12-10961-s001.docx]

| Gene | Forward primer (5'-3') | Reverse primer (5'-3') |
| --- | --- | --- |
| β-actin | CACCATTGGCAATGAGCGGTTC | AGGTCTTTGCGGATGTCCACGT |
| NSD3 | CAGACGTTTCTGATGTGCAGTCC | CTCCAGGTGAAAGTGTTTGCAGC |
| CKB | GGCAAGCATGAGAAGTTCTCGG | ACCAGCTCCACCTCTGAGAAGC |
| GADD45G | CGTCTACGAGTCAGCCAAAGTC | CGATGTCGTTCTCGCAGCAGAA |
| SCAND1 | CGGCAGTTCCGCTACCAGGAT | TCTTGCACCAGCATCTCCACGA |
| ADAM28 | GTACTGTCGCAGAGTGGATGAC | GTCACTATCCGTCCTTTCCAGG |
| ADAM9 | CTTGCTGCGAAGGAAGTACCTG | CACTCACTGGTTTTTCCTCGGC |
| BIRC3 | GCTTTTGCTGTGATGGTGGACTC | CTTGACGGATGAACTCCTGTCC |
| CXCL5 | CAGACCACGCAAGGAGTTCATC | TTCCTTCCCGTTCTTCAGGGAG |
| DUOX2 | CAATGGCTACCTGTCCTTCCGA | GTCCTTGGAGAGGAAGCCATTC |
| GABRP | ATCCGCCTCTTCTCCAATGGCA | TCAGCCAGGTGAACTCCACATC |
| ITGB6 | TCTCCTGCGTGAGACACAAAGG | GAGCACTCCATCTTCAGAGACG |
| RAB11FIP1 | GCCAGAAAAAGTGCTGCTTCGTC | GGGAAGGGTAAAGTTGACCTGG |

**Supplementary table1** Primers used for real-time PCR assay.

**Supplementary Table 2** Abbreviations of human cancers employed in the manuscript.

| Abbreviations | Full name |
| --- | --- |
| ACC | Adrenocortical carcinoma |
| BLCA | Bladder urothelial carcinoma |
| BRCA | Breast invasive carcinoma |
| CESC | Cervical squamous cell carcinoma and endocervical adenocarcinoma |
| CHOL | Cholangiocarcinoma |
| COAD | Colon adenocarcinoma |
| DLBC | Lymphoid neoplasm diffuse large B-cell lymphoma |
| ESCA | Esophageal carcinoma |
| GBM | Glioblastoma multiforme |
| HNSC | Head and neck squamous cell carcinoma |
| KICH | Kidney chromophobe |
| KIRC | Kidney renal clear cell carcinoma |
| KIRP | Kidney renal papillary cell carcinoma |
| LAML | Acute myeloid leukemia |
| LGG | Brain lower grade glioma |
| LIHC | Liver hepatocellular carcinoma |
| LUAD | Lung adenocarcinoma |
| LUSC | Lung squamous cell carcinoma |
| MESO | Mesothelioma |
| OV | Ovarian serous cystadenocarcinoma |
| PAAD | Pancreatic adenocarcinoma |
| PCPG | Pheochromocytoma and paraganglioma |
| PRAD | Prostate adenocarcinoma |
| READ | Rectum adenocarcinoma |
| SARC | Sarcoma |
| SKCM | Skin cutaneous melanoma |
| STAD | Stomach adenocarcinoma |
| TGCT | Testicular germ cell tumors |
| THCA | Thyroid carcinoma |
| THYM | Thymoma |
| UCEC | Uterine corpus endometrial carcinoma |
| UCS | Uterine carcinosarcoma |
| UVM | Uveal melanoma |

**
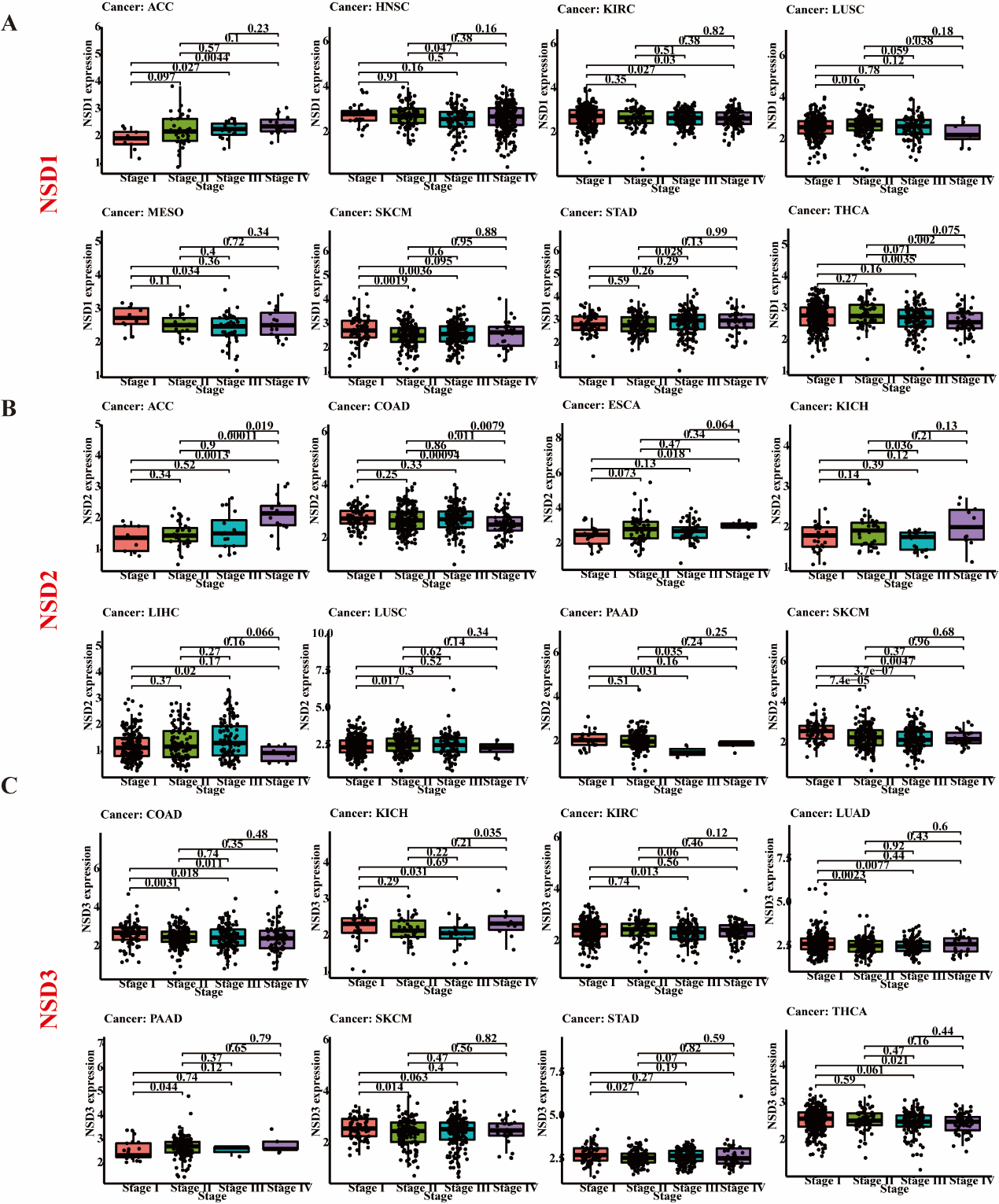
**

**Supplementary Figure 1** The relationship between tumor stage and expression level of NSD1 (A), NSD2 (B), NSD3 (C) in pan-cancer using TCGA datasets. P<0.05 was considered statistically significant.

**
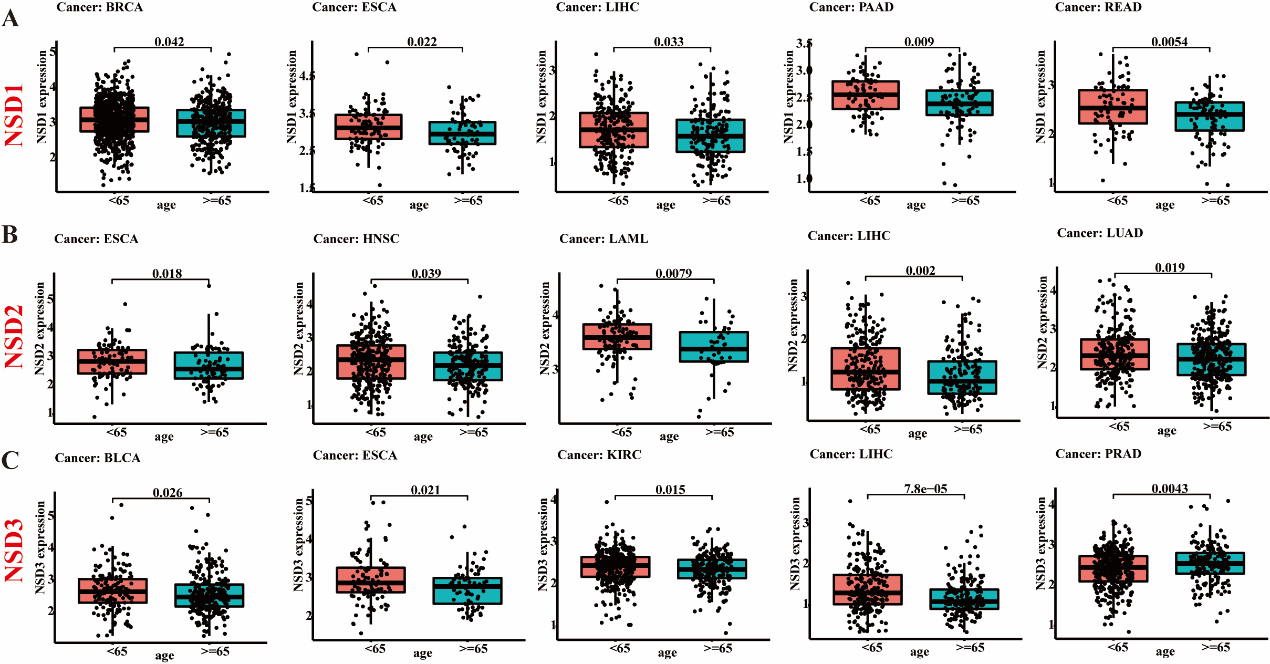
**

**Supplementary Figure 2** The relationship between patient age and expression level of NSD1 (A), NSD2 (B), NSD3 (C) in pan-cancer using TCGA datasets. P<0.05 was considered statistically significant.

**
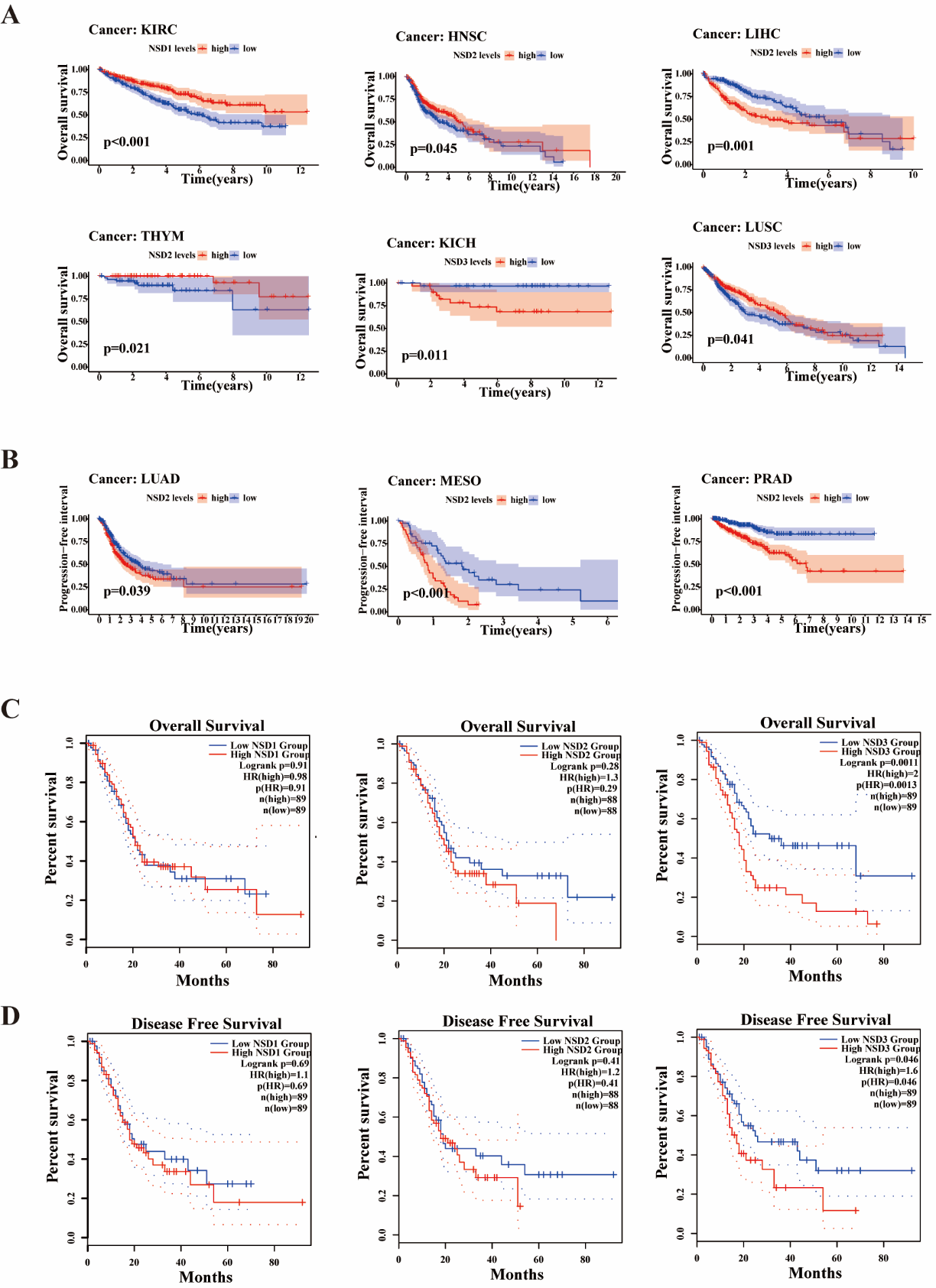
**

**Supplementary Figure 3** Correlation between overall survival (A) as well as progression-free survival (B) and NSD genes from TCGA dataset by Kaplan–Meier method and log-rank test. Correlation between overall survival (A) as well as progression-free survival (B) and NSD1, NSD2, NSD3 in PAAD from GEPIA2 database.

**
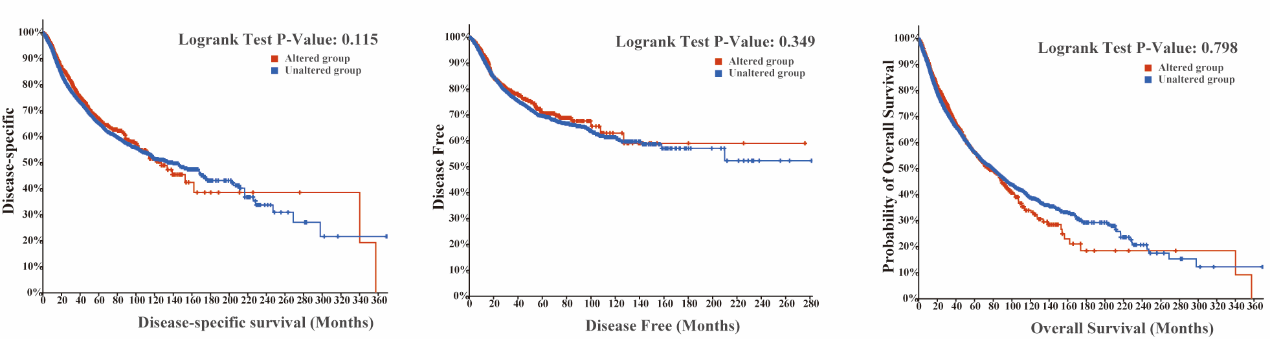
**

**Supplementary Figure 4** the potential correlation between NSD3 alteration and the disease-specific survial, disease-free survival and overall survival of pan-cancer.

**
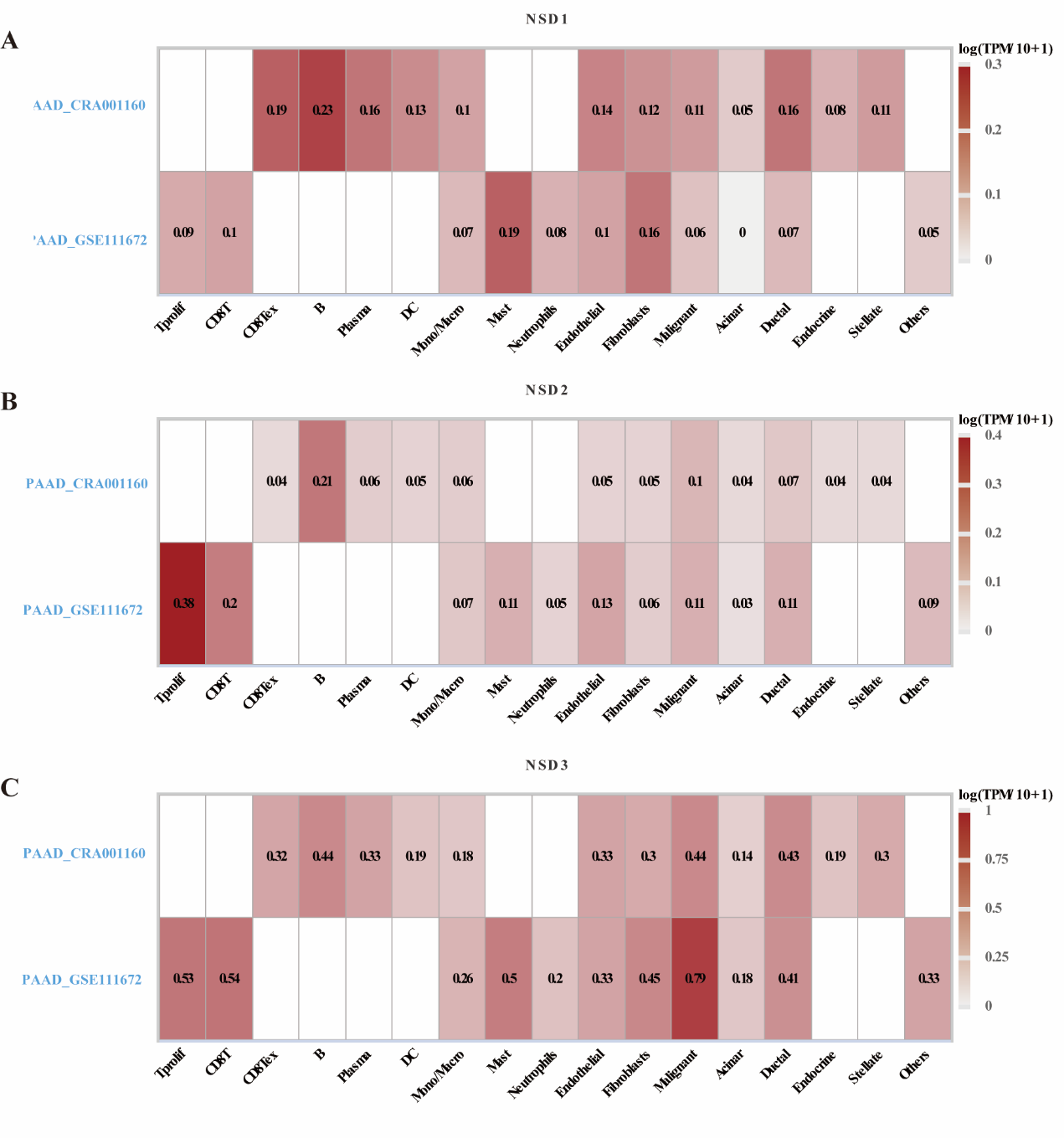
**

**Supplementary Figure 5** The quantified expression level of NSD1 (A), NSD2 (B), NSD3 (C) in different cell types across PAAD datasets, including PAAD_CRA001160 and PAAD_GSE111672, using TISCH database.

**
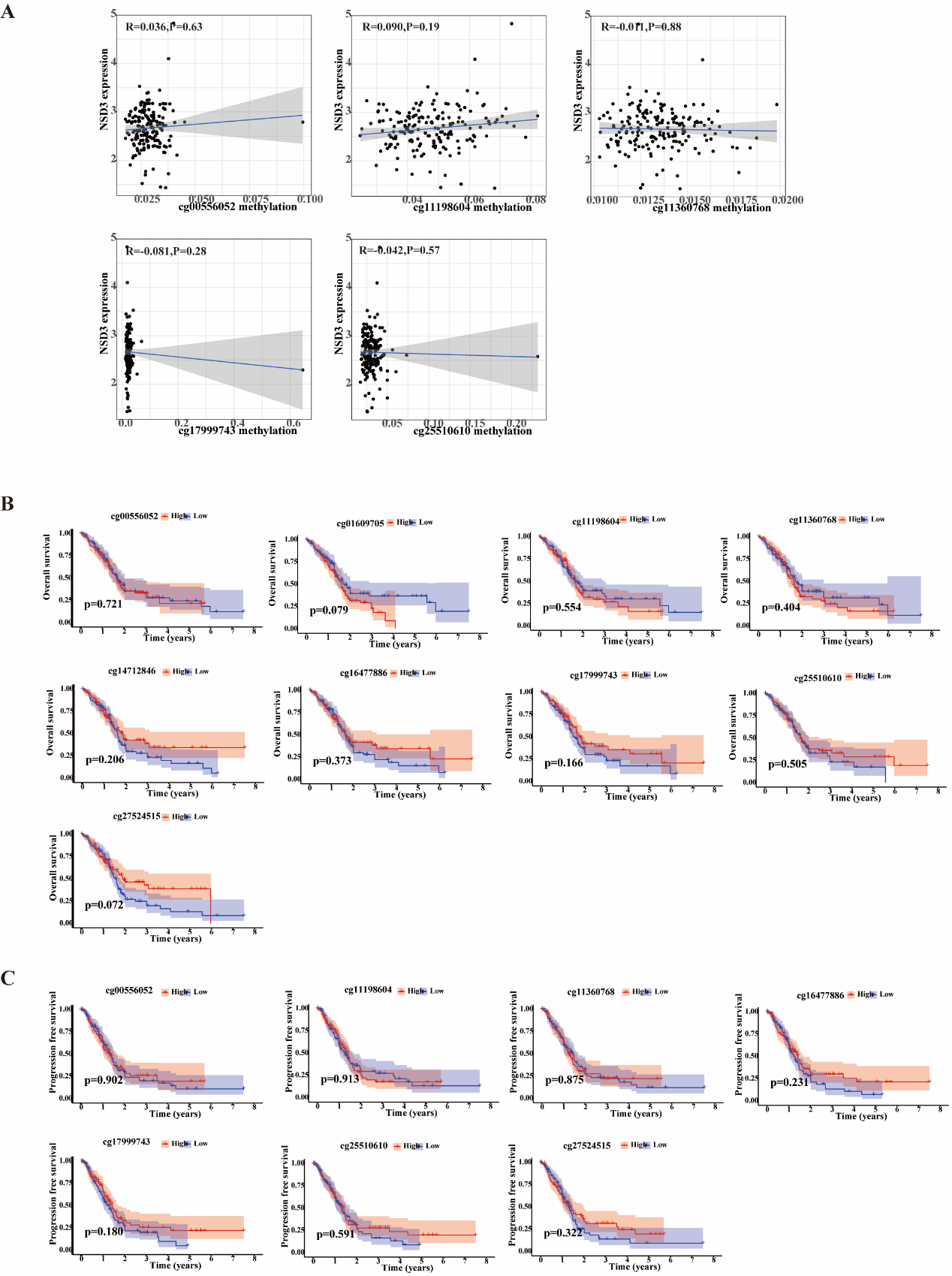
**

**Supplementary Figure 6** The relationship between methylation fo NSD3 promoter and patient survival in PAAD. (A) The relationship between methylation levels of 5 methylation sites and NSD3 expression. (B) The Kaplan–Meier survival curves of overall survival (OS) with methylation levels of 9 methylation sites using log-rank test. (C) The Kaplan–Meier survival curves of progression-free survival (PFS) with methylation levels of 7 methylation sites using log-rank test.
